# Biomechanical and clinical comparison of a novel dual-wing locking plate versus the PHILOS plate for neer three- and four-part proximal humeral fractures: a finite element analysis combined with a retrospective cohort study

**DOI:** 10.3389/fbioe.2026.1865863

**Published:** 2026-08-19

**Authors:** Jianhua Ji, Qinggang Zhao, Sixing Wei, Xingxi Hu, Yongcheng Deng, Zhong Chen, Jinyi Gu, Changshun Chen

**Affiliations:** 1 Department of Orthopaedic and Trauma Surgery, Affiliated Hospital of Yunnan University, Kunming, China; 2 Clinical Laboratory of Affiliated Hospital of Yunnan University, Kunming, China

**Keywords:** bone healing, dual-wing locking plate, finite element analysis, PHILOS plate, proximal humeral fracture, retrospective cohort study, tuberosity fixation

## Abstract

**Background:**

Neer three- and four-part proximal humeral fractures treated with conventional PHILOS plating are associated with high complication rates, primarily due to tuberosity re-displacement and calcar screw cut-out. We developed a novel dual-wing locking plate featuring bilateral tuberosity-capturing wing extensions and an omnidirectional ball-head calcar screw. This study compared its biomechanical performance and clinical outcomes with those of the PHILOS plate.

**Methods:**

A three-dimensional finite element model was reconstructed from CT data using Mimics 21.0, Geomagic 2021, and Rhino 7.0, and analyzed in ANSYS 2022R1 under four shoulder abduction angles (0°, 30°, 60°, 90°) with angle-specific physiological rotator cuff loading. Outcome measures included von Mises stress and displacement in the plate and bone, as well as implant stress distribution. Clinically, we reviewed 43 consecutive patients with Neer three- or four-part fractures treated between January 2023 and December 2024: 19 with the dual-wing plate (Group A) and 24 with the PHILOS plate (Group B). Recorded parameters included Visual Analog Scale score, Constant–Murley score shoulder range of motion, fracture union, and complications.

**Results:**

Across all abduction angles, peak bone von Mises stress and maximum bone displacement were comparable between constructs (differences <2%). The principal biomechanical difference was stress distribution: the PHILOS plate concentrated peak stress at the proximal calcar screw-plate junction at every angle, whereas the dual-wing plate shifted this concentration to the wing-main plate interface, away from the articular region. Peak plate stress was similar at 0° (42.9 vs. 43.3 MPa) and 30° (59.4 vs. 60.6 MPa), but diverged at 60° (67.2 vs. 82.3 MPa) and 90° (68.4 vs. 108.7 MPa), reflecting progressive wing loading. Clinically, Group A initiated passive pendulum exercises 3.7 days earlier (6.26 ± 0.73 vs. 9.96 ± 1.37 days; p < 0.001), had lower VAS at 1 month (p = 0.005), higher CMS at 1 month (p = 0.003) and 2 months (p < 0.001). No tuberosity nonunion or malunion occurred in Group A versus three cases in Group B.

**Conclusion:**

The dual-wing plate does not reduce overall bone stress or displacement but fundamentally redirects implant stress concentration from the calcar screw region to the wing-plate interface. This redistribution was associated with earlier passive pendulum exercise initiation and a lower observed rate of tuberosity complications in this small cohort.

## 1 Introduction

Proximal humeral fractures account for approximately 5%–6% of all fractures, ranking third in frequency among fragility fractures in older adults after hip and distal radius injuries (Passaretti et al., 2017; Khatib et al., 2014). The incidence among patients aged 65 years or older rose by 28% between 2010 and 2020 (Khatib et al., 2014), reflecting the ongoing demographic shift toward an older population. Among the various fracture patterns defined by Neer (1970), three- and four-part fractures are the most surgically demanding, involving comminution of both tuberosities, disruption of the rotator cuff insertions, and loss of medial calcar support. Locking plates, and the PHILOS system in particular, have become the standard implant for displaced three- and four-part fractures, with prospective multicenter series reporting satisfactory outcomes in the majority of patients (Brunner et al., 2009; Sproul et al., 2011; Südkamp et al., 2009); however, complication rates of 15%–35% have been reported in systematic reviews, driven primarily by varus collapse, screw cut-out, and tuberosity malunion (Thanasas et al., 2009).

The shoulder is the most mobile joint in the body (Goldstein, 2004). During arm abduction, the supraspinatus anchors the humeral head against the glenoid while the deltoid generates the abduction moment; the infraspinatus and teres minor contract during external rotation, and the subscapularis during internal rotation (Keating et al., 1993). When the greater tuberosity fractures, these muscles — particularly the supraspinatus, infraspinatus, and teres minor — pull the fragment superiorly. Failure to achieve anatomical reduction shortens the muscle-tendon unit, reduces rotator cuff contractile force, and predisposes to secondary subacromial impingement and chronic shoulder pain. Lesser tuberosity malunion, driven by the subscapularis, limits internal rotation and produces persistent discomfort (Hardeman et al., 2012; Gruson et al., 2008). Handschin et al. (Handschin et al., 2008) documented that delayed or malunited tuberosity fragments consistently impair rotator cuff function and generate lasting disability. Kettler et al. (2006) demonstrated that anatomical tuberosity reduction is among the strongest predictors of Constant-Murley score, reinforcing that anatomical healing of these fragments is the key determinant of functional outcome.

Despite the clinical importance of tuberosity fixation, conventional implant options remain inadequate. Isolated screws risk further comminution; tension band wiring provides good fixation strength but risks iatrogenic surgical neck fracture; suture fixation offers relative stability at best, with Braunstein et al. (2007) demonstrating that suture-based constructs lack the mechanical rigidity needed for safe early rehabilitation. The PHILOS plate (DePuy Synthes) addresses head-shaft fixation effectively but cannot directly envelop displaced tuberosity fragments; supplementary suture repair is typically required (Badman et al., 2011; Voigt et al., 2009). Voigt et al. (Voigt et al., 2009) showed that additive suture cerclages provide no meaningful improvement in fracture stabilization in biomechanical testing. Various augmentation strategies have been proposed to address medial column deficiency, including fibular strut allograft (Verma et al., 2025) and cement augmentation of calcar screws (She et al., 2023), yet neither directly resolves the problem of tuberosity fragment fixation. Calcar screw cut-out—driven by stress concentration at the proximal screw-plate junction—remains the leading mechanical failure mode in published clinical series (Varga et al., 2020; Liu et al., 2024).

Finite element analysis (FEA) provides a reproducible platform for comparing implant designs under controlled loading, allowing evaluation of stress distribution and implant mechanics across physiological conditions (Mischler et al., 2020). Prior proximal humeral FEA studies have identified calcar screw placement and the pattern of stress concentration at the plate-bone interface as the principal determinants of mechanical failure risk (Feerick et al., 2013; He et al., 2017; Jabran et al., 2019; Liu et al., 2024; Lv et al., 2024; Mischler et al., 2020; Schader et al., 2022; Zdero et al., 2024). Most of these studies apply a single loading condition; a multi-angle analysis spanning the full arc of early rehabilitation is more clinically informative.

We developed a dual-wing locking plate (Patent No. ZL202022885149.6; Suzhou Aider Technology, China) targeting two specific limitations of the PHILOS system: (1) snap-lock bilateral wing extensions allow auxiliary locking mini-plates to be secured directly to each tuberosity fragment without repositioning the main plate; and (2) an omnidirectional ball-head calcar screw permits ±15° angulation in both the coronal and sagittal planes, enabling individualized placement into the densest inferomedial bone regardless of native neck-shaft angle or fracture comminution. We combined a four-angle FEA with a retrospective cohort study to determine whether these mechanical differences translate into demonstrably better clinical outcomes.

## 2 Materials and methods

### 2.1 Finite element analysis

#### 2.1.1 CT acquisition and three-dimensional reconstruction

**FIGURE 1:**
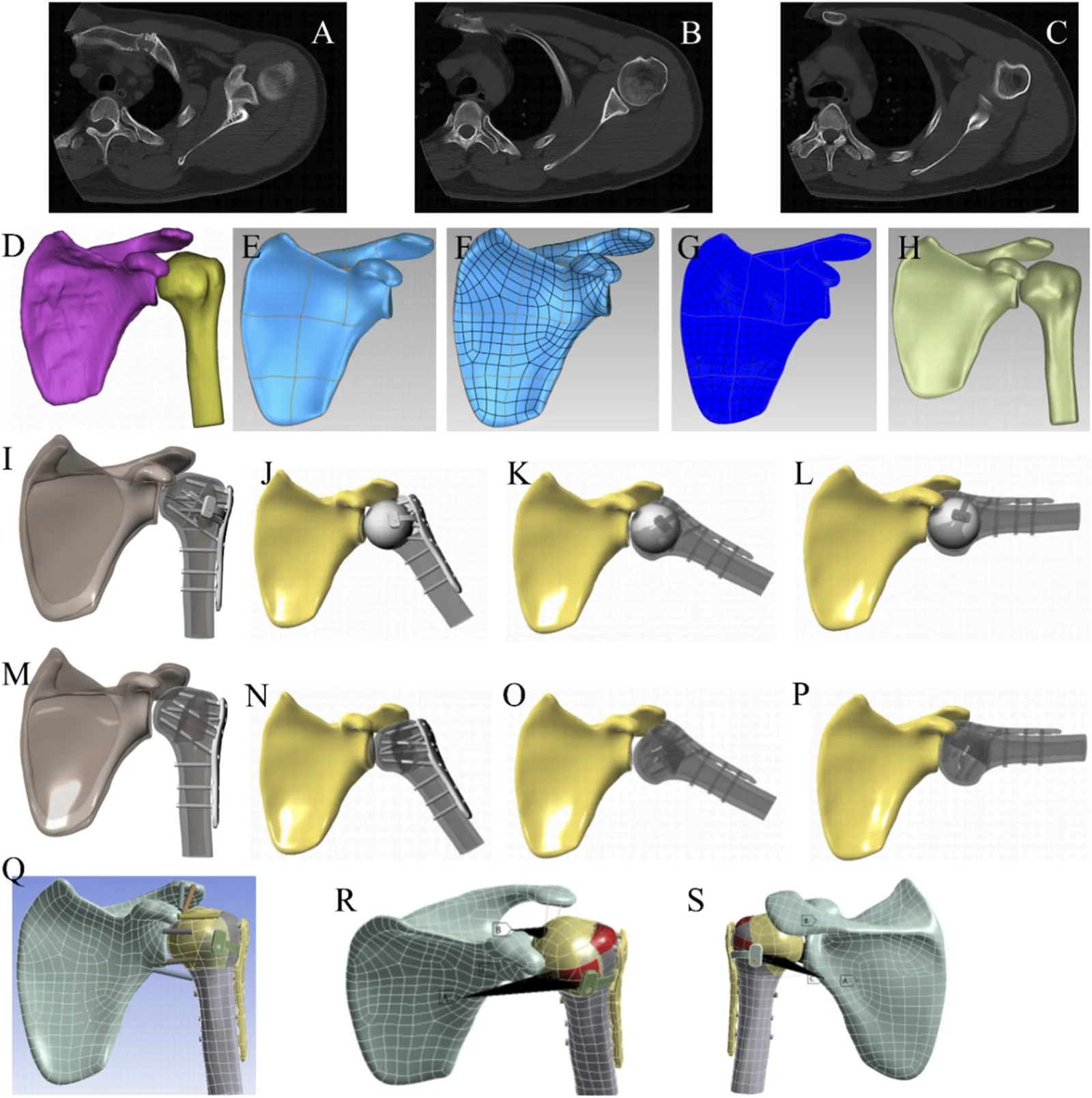
FEA model construction and loading configuration. **(A–C)** representative axial CT slices at the proximal head **(A)**, anatomical neck **(B)**, and surgical neck **(C)**. **(D–H)** three-dimensional reconstruction pipeline in Geomagic 2021: initial Mimics surface mesh **(D)**, smoothing and de-noising **(E)**, contour line placement for NURBS fitting **(F)**, surface patch construction **(G)**, and completed NURBS solid **(H)**. **(I)** dual-wing plate assembled on the four-part fracture model with full shoulder geometry; **(J–L)** dual-wing construct at 0°, 30°, and 60° abduction; **(M)** PHILOS plate on an identical fracture model; **(N–P)** PHILOS construct at 0°, 30°, and 60° abduction. **(Q)** finite element mesh of the complete assembly with ligament beam elements; **(R,S)** rotator cuff loading configuration at 90° abduction, anterior view **(R)** and rear view **(S)**, showing coupling element force vectors applied at supraspinatus (superior facet), infraspinatus + teres minor (posterior facet), and subscapularis (lesser tuberosity) insertion centroids.

Shoulder CT data were acquired from a healthy male volunteer with no history of shoulder pathology (written informed consent obtained; institutional ethics approval granted). Scanning was performed on a 32-slice helical CT scanner (Siemens, Germany) at 2 mm slice thickness. DICOM images were imported into Mimics 21.0 (Materialise, Belgium); cortical and cancellous bone were segmented using a threshold of 600 Hounsfield units. The resulting surface mesh was transferred to Geomagic 2021 (3D Systems, United States) for surface smoothing, de-noising, and NURBS surface fitting. Solid bodies were generated for the humerus (cortical and cancellous compartments separately), the scapula, and the five principal glenohumeral ligaments. The reconstruction pipeline is illustrated in Figures 1A–H.

#### 2.1.2 Model assembly, fracture simulation, and ligament construction

Solid models were imported into Rhino 7.0 for assembly and interference checking. A standard Neer four-part fracture was created with three osteotomy planes — at the surgical neck, at the base of the greater tuberosity, and at the base of the lesser tuberosity — leaving a 1-mm anatomically reduced gap at each interface. Three-dimensional CAD models of both implants were constructed from direct measurements (Figures 1I–P). The five glenohumeral ligaments were modeled as tension-only circular cross-section beam elements (diameter 2 mm), connecting anatomical origins to insertion points (Figures 1Q–S). The assembled models are shown in Figure 1.

#### 2.1.3 Mesh generation and material properties

The assembly was imported into ANSYS 2022R1 (Ansys Inc., United States) and meshed with ten-node tetrahedral elements (1.0 mm for bone, 0.8 mm for implants); mesh convergence confirmed at <3% change in peak stress between refinements. Contact conditions: screw–bone and plate–screw interfaces bonded; dual-wing extension–main plate frictionless; fracture interfaces frictionless; cortical–cancellous bonded. Material properties are provided in Supplementary Table S1.

#### 2.1.4 Rotator cuff loading and boundary conditions

The scapula was fully constrained. Four abduction angles (0°, 30°, 60°, 90°) were modeled by rotating the humeral assembly about the glenoid center. Rotator cuff forces were applied as concentrated loads following the method of Apreleva et al. (Apreleva et al., 2000), with loading directions at each angle per Keating et al. (Keating et al., 1993; Apreleva et al., 2000). Force magnitudes at each abduction angle are given in Supplementary Table S2. The loading configuration is shown in Figures 1R–S.

#### 2.1.5 Outcome variables

1. Von Mises stress: peak values (MPa) in the plate and bone, with full contour distribution maps.
2. Displacement: maximum resultant displacement (mm) of the plate and bone.
3. Stress distribution pattern: qualitative assessment of the site of peak stress concentration within each implant and its anatomical relationship to the calcar and articular surface.

### 2.2 Clinical study

#### 2.2.1 Study design and patient selection

We retrospectively reviewed all consecutive patients who underwent ORIF for a Neer three- or four-part proximal humeral fracture at the Affiliated Hospital of Yunnan University between January 2023 and December 2024. The study was approved by the institutional ethics committee (Approval No. 2023100), and all procedures complied with the Declaration of Helsinki. Written informed consent was obtained from each participant.

Inclusion criteria: (1) Neer three- or four-part fracture confirmed on radiographs and CT; (2) age 18–80 years; (3) fresh fracture (≤2 weeks from injury); (4) ORIF with dual-wing or PHILOS plate; (5) minimum 12-month follow-up with complete records.

Exclusion criteria: (1) severe systemic comorbidities precluding surgery; (2) concomitant ipsilateral neurovascular injury or polytrauma; (3) pathological or old fracture (>2 weeks from injury); (4) pre-existing ipsilateral shoulder dysfunction or prior shoulder surgery; (5) incomplete records or loss to follow-up before 12 months.

After applying these criteria, 43 patients were included: 19 in Group A (dual-wing) and 24 in Group B (PHILOS). Implant selection was determined jointly by the operating surgeon and patient based on fracture morphology and bone quality.

#### 2.2.2 Implant specifications

The dual-wing locking plate (Suzhou Aider Technology, China) is made of pure titanium (TA3) and consists of a main plate body and detachable wing mini-plates. The proximal part of the main plate carries a snap-lock interface on each side; depending on the fixation needs, a wing mini-plate can be inserted into one or both interfaces, and after assembly the construct takes the form of two wings that directly wrap and compress the greater and lesser tuberosity fragments. The calcar screw hole at the distal part of the main plate uses a ball-head omnidirectional design, which allows the screw axis to be adjusted by ±15° in both the coronal and the sagittal plane, so that the surgeon can place the screw into the most effective supporting region according to the individual neck-shaft angle and fracture reduction. The plate is intended for internal fixation of Neer three- and four-part proximal humeral fractures: the omnidirectional calcar screw provides individualized support of the medial column, and the wing mini-plates provide stable fixation of the free tuberosity fragments (Supplementary Figure S1).

#### 2.2.3 Surgical technique

All procedures were performed under general anesthesia in the beach-chair position via a standard deltopectoral approach. Tuberosity fragments were provisionally reduced with K-wires and heavy non-absorbable sutures (No. 2 Ethibond) pre-placed through the rotator cuff tendons.

Group B (PHILOS): Plate positioned at the standard anterolateral location, approximately 5–8 mm distal to the greater tuberosity apex. Six proximal locking screws were placed into the humeral head; three distal screws secured the shaft. Tuberosity reduction was maintained with heavy sutures through the dedicated plate suture holes.

Group A (dual-wing plate): Main plate body positioned identically to PHILOS. The omnidirectional ball-head calcar screw was directed under fluoroscopy toward the posteromedial trabecular column, with angulation adjusted up to ±15° in each plane to achieve optimal inferomedial support. Wing extensions were assembled into the snap-lock interfaces according to fracture configuration — one or both wings deployed as needed — and secured with three dedicated locking screws per wing, providing direct rigid fixation of each tuberosity fragment.

#### 2.2.4 Postoperative rehabilitation

All patients followed an identical standardized rehabilitation protocol, supervised by the same senior physiotherapist who was not involved in surgery or group allocation. Postoperative weeks 0–6: The arm was immobilized in a sling. From postoperative day 2, active distal limb exercises (finger flexion/extension, wrist flexion/extension, and elbow flexion/extension) were initiated, performed in 3 sets of 15–20 repetitions daily under physiotherapist guidance. Based on individual pain tolerance, passive pendulum exercises of the shoulder were started within the first two postoperative weeks; active shoulder motion was strictly prohibited during this entire phase. Postoperative weeks 4–7: After radiographic confirmation of initial fracture healing (callus formation or stable alignment), the sling was removed, and passive and active-assisted shoulder exercises (forward flexion, abduction, internal/external rotation, and pendulum motions) were commenced. Postoperative weeks 8–12: Gradually transition to full active shoulder motion and begin progressive resistive strengthening of the peri-scapular and rotator cuff muscles.

#### 2.2.5 Outcome measures

Perioperative data: operative time, blood loss, time to first passive pendulum exercise, wound healing, and hospital stay. Clinical outcomes at 1, 2, 3, 6 months, and final follow-up: VAS (0–10), CMS (0–100), and active ROM in four planes. Fracture union (bridging callus on ≥3 cortices) classified as ≤8, 8–12, or >12 weeks. Complications recorded: DVT, screw cut-out, tuberosity nonunion or malunion, AVN, wound infection, and neurovascular injury. Standardized radiographic follow-up was performed: anteroposterior and lateral radiographs of the scapula were obtained at 1, 3, and 6 months after surgery and at the final follow-up. Two independent observers who were not involved in the surgery assessed neck-shaft angle, loss of reduction, varus collapse, screw penetration or cut-out, humeral head height, tuberosity displacement or migration, tuberosity union, and avascular necrosis; these results are reported as reduction quality and complications.

#### 2.2.6 Statistical analysis

Continuous data are presented as mean ± SD. The Shapiro-Wilk test assessed normality; normally distributed variables were compared with the independent t-test and non-normally distributed variables with the Mann-Whitney U test. Categorical variables were compared with chi-squared or Fisher exact test. All analyses used SPSS Statistics 26.0 (IBM Corp., Armonk, NY); p < 0.05 was the threshold for significance.

## 3 Results

### 3.1 Finite element analysis

#### 3.1.1 Model construction

The final FEA assembly comprised 311,267 elements and 78,839 nodes. Mesh convergence was confirmed at <3% change in peak von Mises stress between successive refinement levels.

#### 3.1.2 Results at 0° abduction

**FIGURE 2:**
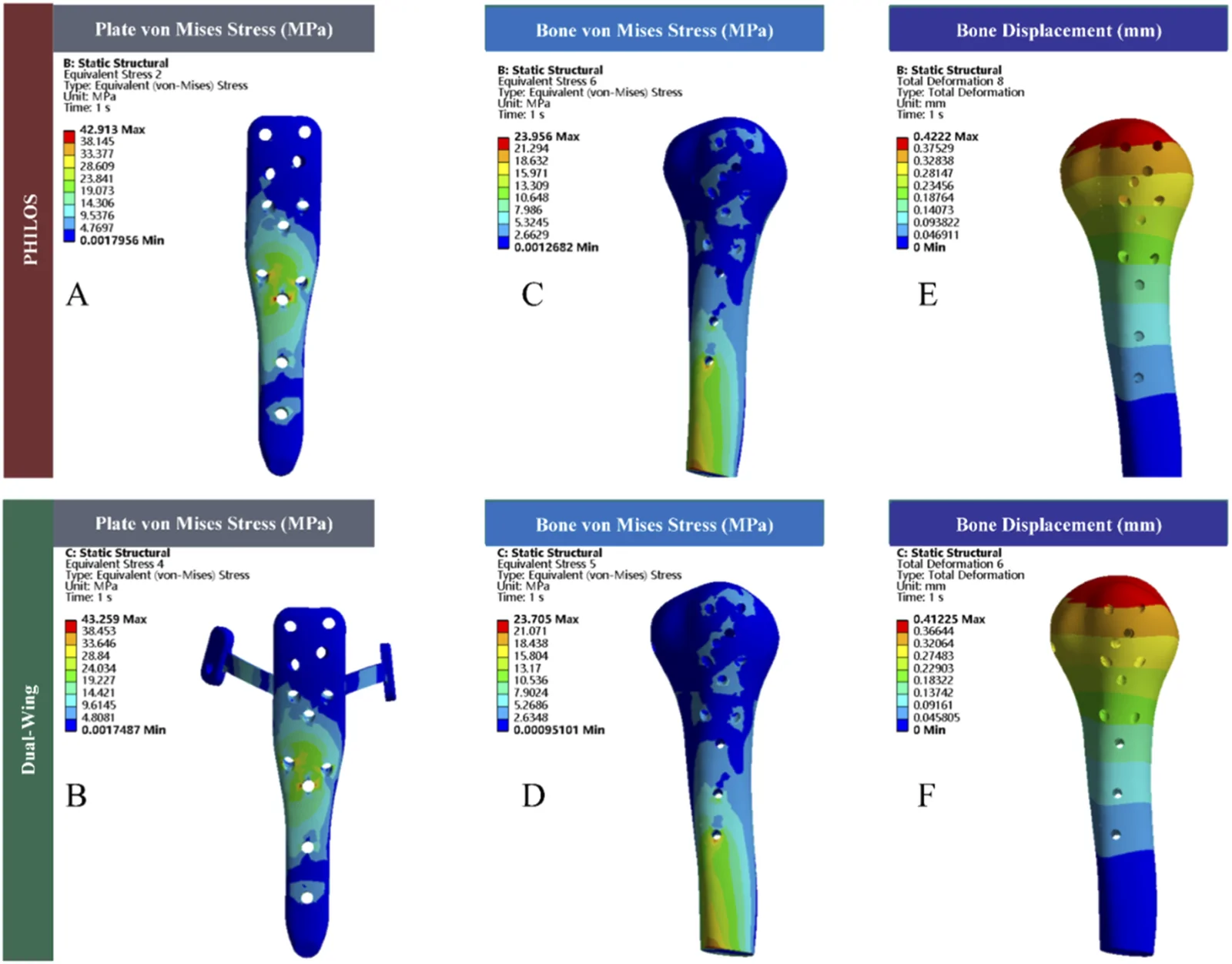
FEA results at 0° abduction. **(A,B)** Plate von Mises stress (MPa) — PHILOS (A) and dual-wing (B); **(C,D)** bone von Mises stress (MPa); **(E,F)** bone displacement (mm). Stress concentration at the proximal calcar screw-plate junction is evident in the PHILOS model (A); the dual-wing model shows distributed loading across both wing extensions and the plate body (B). Screw results provided in Supplementary Figure S2.

At the neutral position, rotator cuff forces are at their minimum (supraspinatus 100 N, subscapularis 60 N, infraspinatus 30 N, teres minor 20 N; Supplementary Table S2). Peak von Mises stress in the PHILOS plate body reached 42.913 MPa, compared with 43.259 MPa in the dual-wing plate — essentially equivalent, with the dual-wing plate carrying marginally higher peak stress owing to load transfer into the wing extensions. Peak bone von Mises stress was 23.956 MPa under PHILOS and 23.705 MPa under the dual-wing construct, virtually identical between groups. Maximum bone displacement was 0.4222 mm (PHILOS) and 0.41225 mm (dual-wing); maximum plate displacement was 0.39419 mm and 0.38509 mm respectively. The stress distribution contour maps show that in the PHILOS model stress concentrated around the proximal calcar screw-plate junction, while in the dual-wing model stress was distributed more broadly across the main plate body and both wing extensions. At this loading level the mechanical difference between constructs is small in absolute terms (Figure 2; Supplementary Figure S2).

#### 3.1.3 Results at 30° abduction

**FIGURE 3:**
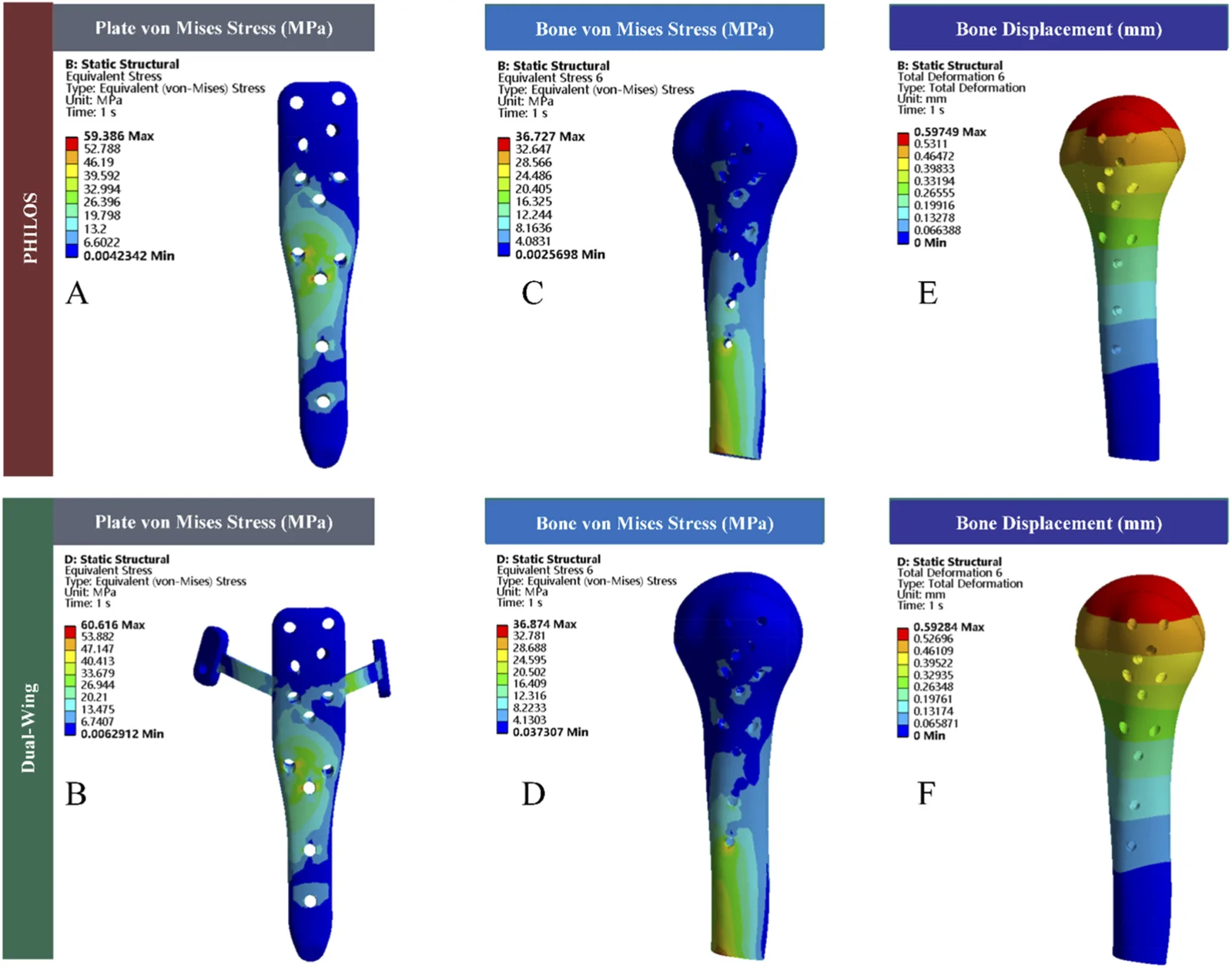
FEA results at 30° abduction. **(A,B)** plate von Mises stress (MPa); **(C,D)** bone stress (MPa); **(E,F)** bone displacement (mm). The wing extension directly resists the supraspinatus deforming force in the dual-wing construct (B). Screw results provided in Supplementary Figure S3.

At 30° abduction, supraspinatus force increases to 120 N and subscapularis to 80 N, substantially raising the deforming load on both tuberosities. Peak plate stress was 59.386 MPa in the PHILOS construct and 60.616 MPa in the dual-wing construct. Peak bone stress rose to 36.727 MPa (PHILOS) and 36.874 MPa (dual-wing), differing by less than 0.5%. Maximum bone displacement was 0.59749 mm (PHILOS) and 0.59284 mm (dual-wing); maximum plate displacement was 0.55563 mm versus 0.55305 mm. The contour maps show continued calcar junction stress concentration in the PHILOS model, while the dual-wing model distributed load across both wings and the main plate shaft. Despite similar peak values, the wing extensions absorbed a portion of the supraspinatus-generated force directly at the greater tuberosity footprint, reducing focal calcar stress concentration even as the global maximum remained comparable (Figure 3; Supplementary Figure S3).

#### 3.1.4 Results at 60° abduction

**FIGURE 4:**
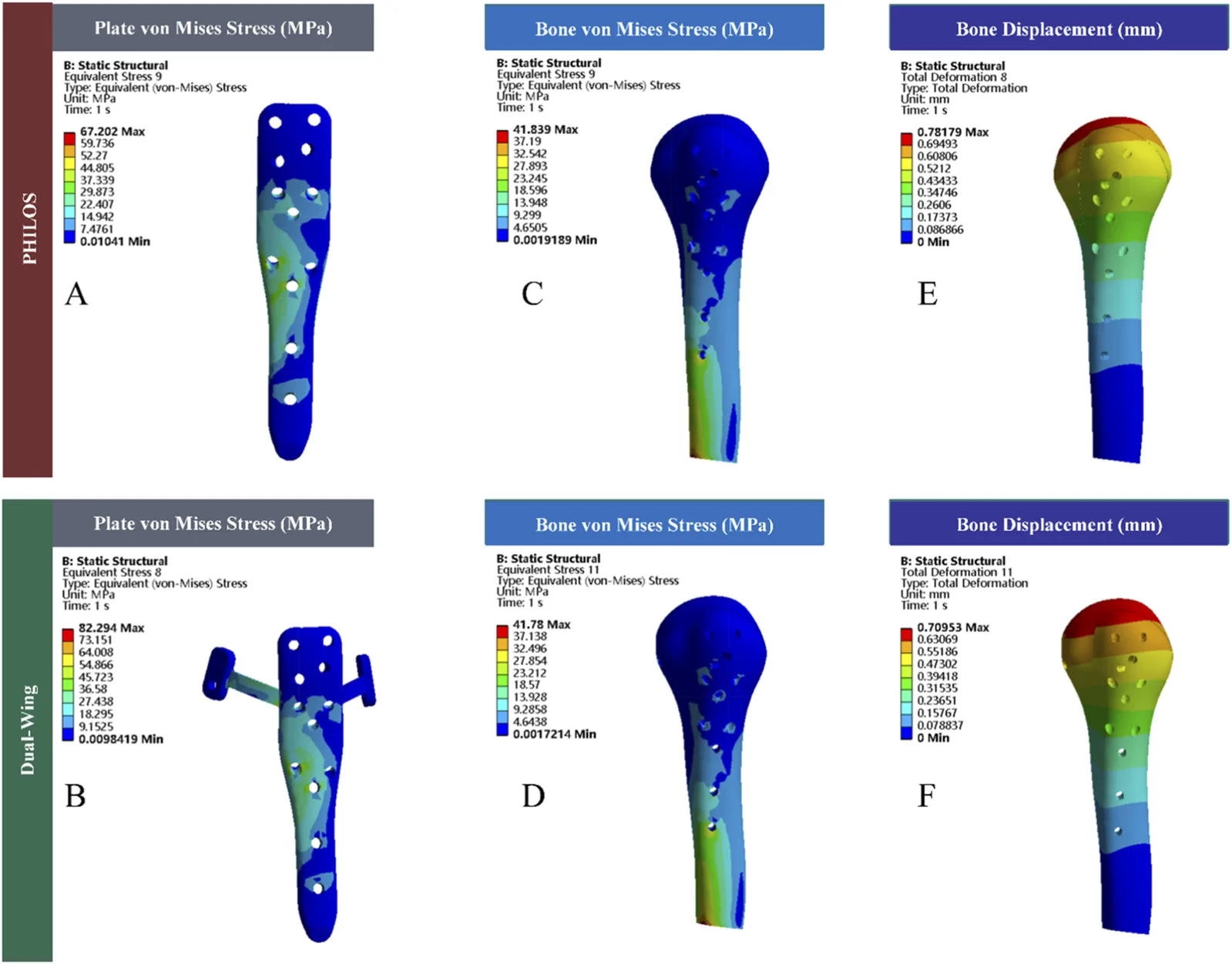
FEA results at 60° abduction. **(A,B)** plate von Mises stress (MPa); **(C,D)** bone stress (MPa); **(E,F)** bone displacement (mm). Peak plate stress is higher in the dual-wing construct (82.3 vs. 67.2 MPa), concentrated at the wing-main plate junction rather than the calcar region. Bone stress and displacement are nearly identical between groups. Screw results provided in Supplementary Figure S4.

At 60° abduction, combined loading reached its highest intensity (supraspinatus 150 N, subscapularis 100 N, infraspinatus 80 N, teres minor 50 N), and the two constructs began to diverge more clearly. Peak plate stress was 67.202 MPa in the PHILOS model, while the dual-wing plate registered 82.294 MPa — 22.5% higher — reflecting concentration of rotator cuff tensile forces at the wing-main plate junction as muscle activation increases. Peak bone von Mises stress was virtually identical: 41.839 MPa (PHILOS) and 41.78 MPa (dual-wing), differing by less than 0.1%. Maximum bone displacement was 0.78179 mm (PHILOS) and 0.70953 mm (dual-wing), a 9.2% reduction. Maximum plate displacement was 0.61249 mm (PHILOS) and 0.64248 mm (dual-wing) (Figure 4; Supplementary Figure S4).

#### 3.1.5 Results at 90° abduction

**FIGURE 5:**
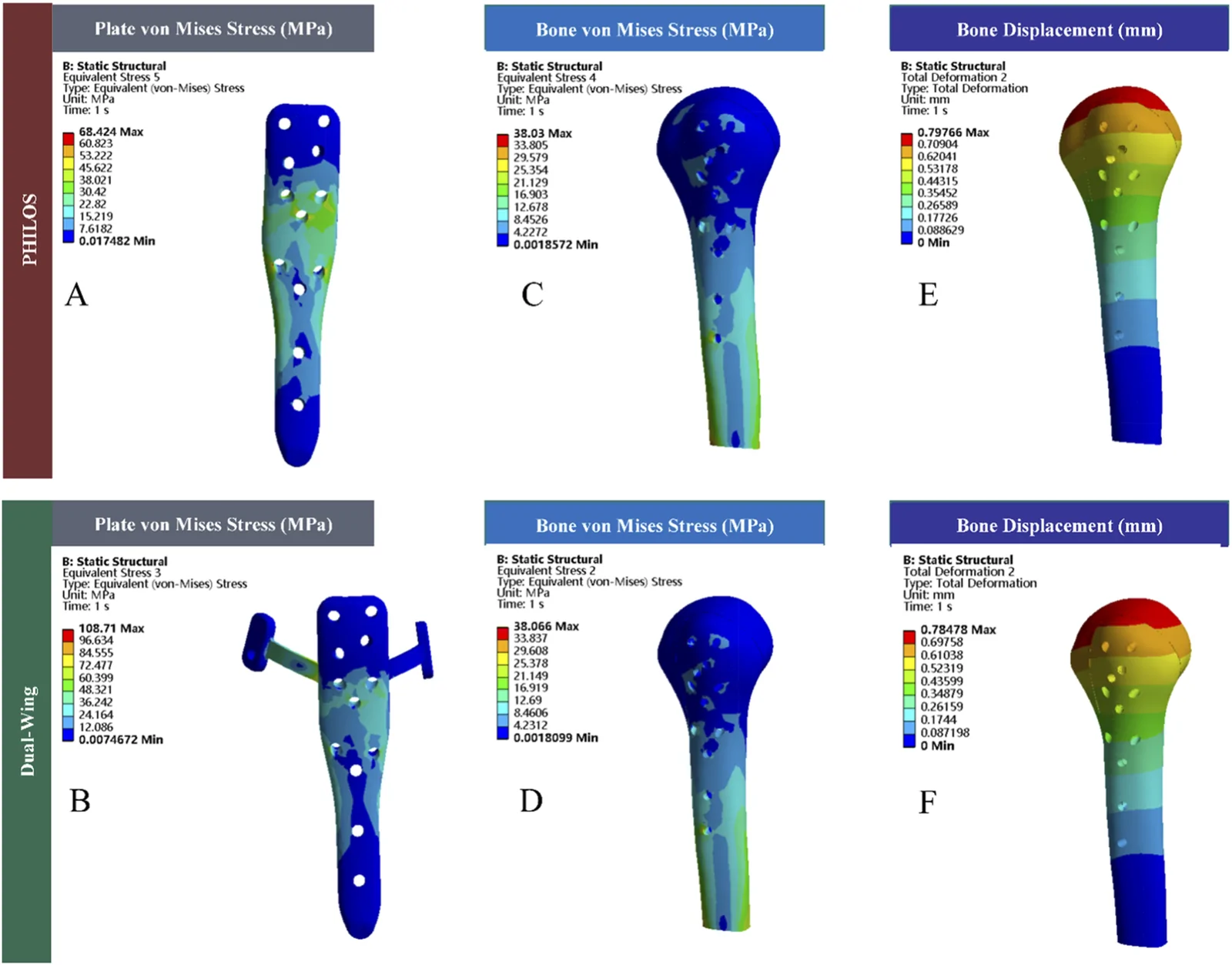
FEA results at 90° abduction. **(A,B)** plate von Mises stress (MPa); **(C,D)** bone stress (MPa); **(E,F)** bone displacement (mm). Peak plate stress is markedly higher in the dual-wing construct (108.7 vs. 68.4 MPa), concentrated at the wing-main plate junction. Bone stress and displacement are essentially equivalent between groups. Screw results provided in Supplementary Figure S5.

At 90° abduction, full rotator cuff co-activation produces maximum mechanical demand (supraspinatus 180 N, subscapularis 120 N, infraspinatus 100 N, teres minor 60 N). The dual-wing plate reached a peak of 108.71 MPa, compared with 68.424 MPa for PHILOS — 58.9% higher in the dual-wing model — reflecting the progressive accumulation of rotator cuff tensile forces through the wing-main plate junction. Peak bone von Mises stress was nearly identical: 38.03 MPa (PHILOS) and 38.066 MPa (dual-wing), differing by less than 0.1%. Maximum bone displacement was 0.79766 mm (PHILOS) and 0.78478 mm (dual-wing), a 1.6% difference. Maximum plate displacement was 0.66523 mm (PHILOS) and 0.76825 mm (dual-wing). The 90° data confirm the pattern established at 60°: the dual-wing plate does not reduce bone-level stress or displacement relative to PHILOS, but shifts peak implant stress away from the calcar screw region and into the wing bodies (Figure 5; Supplementary Figure S5).

### 3.2 Patients and perioperative data

Nineteen patients received the dual-wing plate (Group A) and 24 received PHILOS (Group B). Baseline and perioperative characteristics are summarized in Table 1. Both groups were well matched on all preoperative variables (all p > 0.05). Operative time, blood loss, and fracture reduction quality were similar. Group A had a marginally shorter hospital stay (8.26 vs. 8.88 days; p = 0.040) and began passive pendulum exercises significantly earlier (6.26 ± 0.73 vs. 9.96 ± 1.37 days; p < 0.001). All wounds healed primarily. No augmentation technique (fibular strut graft, bone graft, bone substitute, cement augmentation, or medial suture or cerclage) was used in either group. Medial support was obtained by anatomical reduction of the calcar and inferomedial screw placement alone, and the reduction quality was good to excellent in all cases (Table 1).

**TABLE 1:** Baseline demographic and perioperative characteristics.

Characteristic	Group A (n = 19)	Group B (n = 24)	P value
Age (years), mean ± SD	52.21 ± 15.75	52.88 ± 13.66	0.883
Sex, M/F	10/9	13/11	1.000
BMI (kg/m^2^), mean ± SD	23.46 ± 1.12	23.89 ± 1.17	0.230
Neer fracture type, n (%)	​	​	0.495
3-part	9 (47.4%)	15 (62.5%)	​
4-part	10 (52.6%)	9 (37.5%)	​
Operative time (min)	84.32 ± 8.86	81.88 ± 5.48	0.274
Blood loss (mL)	127.11 ± 19.32	121.25 ± 16.50	0.290
Hospital stay (days)	8.26 ± 1.15	8.88 ± 0.74	0.040
Time to first passive pendulum exercise (days)	6.26 ± 0.73	9.96 ± 1.37	<0.001
Fracture reduction, n (%)	​	​	0.270
Excellent	17 (89.5%)	18 (75.0%)	​
Good	2 (10.5%)	6 (25.0%)	​

Values are mean ± SD, unless stated. Sex: Fisher’s exact; Neer type: chi-squared; continuous variables: independent t-test.

### 3.3 Pain and shoulder function

Group A showed lower VAS at 1 month (2.95 ± 0.71 vs. 3.50 ± 0.51; p = 0.005). CMS was higher in Group A at 1 month (63.84 ± 4.79 vs. 59.92 ± 3.31; p = 0.003) and 2 months (74.63 ± 4.84 vs. 69.92 ± 3.50; p < 0.001). Both groups reached equivalent scores at 6 months and final follow-up (CMS 94.63 ± 1.34 vs. 94.04 ± 1.55; p = 0.196). Results are shown in Table 2.

**TABLE 2:** VAS pain scores and Constant-Murley scores at each follow-up visit (mean ± SD).

Outcome	Group	1 month	2 months	3 months	6 months	Final	P
VAS	A	2.95 ± 0.71	2.42 ± 0.51	1.63 ± 0.50	0.95 ± 0.52	0.63 ± 0.50	0.005
​	B	3.50 ± 0.51	2.62 ± 0.49	1.71 ± 0.46	0.96 ± 0.46	0.58 ± 0.50	​
​	P value	0.005	0.192	0.604	0.942	0.755	​
CMS	A	63.84 ± 4.79	74.63 ± 4.84	85.95 ± 4.67	91.95 ± 3.34	94.63 ± 1.34	0.003
​	B	59.92 ± 3.31	69.92 ± 3.50	83.50 ± 3.74	91.54 ± 3.30	94.04 ± 1.55	​
​	P value	0.003	<0.001	0.063	0.692	0.196	​

P values shown per row; summary P indicates timepoint of largest between-group difference; CMS, Constant-Murley scores.

### 3.4 Range of motion

Shoulder abduction was significantly greater in Group A at 1 month (74.47 ± 4.38° vs. 71.46 ± 3.75°; p = 0.021) and 2 months (91.58 ± 6.25° vs. 87.92 ± 3.88°; p = 0.033). External rotation favored Group A at 2 months (p = 0.022) and 3 months (p = 0.047). Internal rotation was marginally better in Group A at 1 month (p = 0.023). Forward flexion did not differ significantly at any timepoint. All ROM parameters are converged by 6 months (Table 3).

**TABLE 3:** Shoulder range of motion at each follow-up visit (degrees, mean ± SD).

ROM (°)	Grp	1 month	2 months	3 months	6 months	Final
Forward flexion	A	84.47 ± 4.68	114.47 ± 7.05	128.68 ± 4.67	146.05 ± 5.16	159.21 ± 5.07
​	B	84.17 ± 3.51	113.12 ± 4.38	128.54 ± 3.45	145.62 ± 4.25	158.75 ± 4.23
​	P	0.808	0.418	0.910	0.769	0.747
Abduction	A	74.47 ± 4.38	91.58 ± 6.25	112.89 ± 7.13	151.05 ± 4.88	160.53 ± 3.69
​	B	71.46 ± 3.75	87.92 ± 3.88	110.62 ± 6.48	150.62 ± 5.95	160.83 ± 4.34
​	P	0.021	0.033	0.256	0.797	0.820
Internal rotation	A	39.74 ± 4.85	56.84 ± 6.71	73.68 ± 7.04	83.42 ± 5.01	88.42 ± 2.39
​	B	37.08 ± 2.52	54.38 ± 3.06	70.62 ± 4.50	83.12 ± 3.23	88.12 ± 3.23
​	P	0.023	0.079	0.107	0.817	0.749
External rotation	A	18.42 ± 4.43	44.21 ± 5.07	70.26 ± 4.24	81.58 ± 3.36	86.58 ± 3.36
​	B	16.25 ± 3.38	40.62 ± 4.96	67.29 ± 5.10	80.42 ± 3.88	86.67 ± 3.51
​	P	0.089	0.022	0.047	0.296	0.941

Between-group P values given in the P row for each plane of motion.

### 3.5 Fracture union and complications

Overall fracture union time did not differ significantly (p = 0.307), though delayed union (>12 weeks) was more frequent in Group B (29.2% vs. 10.5%). Two patients in Group A developed complications (one DVT, one AVN) versus seven in Group B (two DVT, two AVN, three tuberosity nonunion or malunion). No tuberosity nonunion or malunion occurred in Group A (0% vs. 12.5%; p = 0.243). No wound infections, screw cut-outs, dislocations, or periprosthetic fractures occurred in either group (Table 4).

**TABLE 4:** Fracture union and complications.

Variable	Group A (n = 19)	Group B (n = 24)	P value
Union time, n (%)	​	​	0.307
≤8 weeks	6 (31.6%)	5 (20.8%)	​
8–12 weeks	11 (57.9%)	12 (50.0%)	​
>12 weeks	2 (10.5%)	7 (29.2%)	​
Total complications, n (%)	2 (10.5%)	7 (29.2%)	0.258
Deep vein thrombosis	1 (5.3%)	2 (8.3%)	1.000
Tuberosity nonunion/malunion	0 (0%)	3 (12.5%)	0.243
Avascular necrosis of humeral head	1 (5.3%)	2 (8.3%)	1.000

Fisher’s exact test for all categorical comparisons.

### 3.6 Representative cases

**FIGURE 6:**
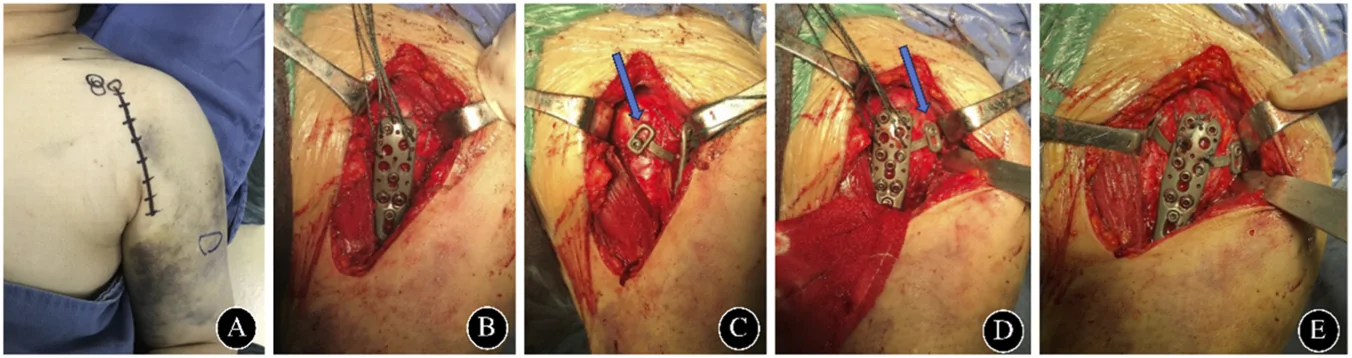
Surgical technique of the dual-wing locking plate. **(A)** Deltopectoral approach; **(B)** main plate body insertion; **(C)** lesser tuberosity wing plate into the medial snap-lock; **(D)** greater tuberosity wing plate into the lateral snap-lock; **(E)** completed bilateral construct.

Two representative cases illustrate the typical clinical and radiological course. The surgical technique of the dual-wing plate is shown in Figure 6.

**FIGURE 7:**
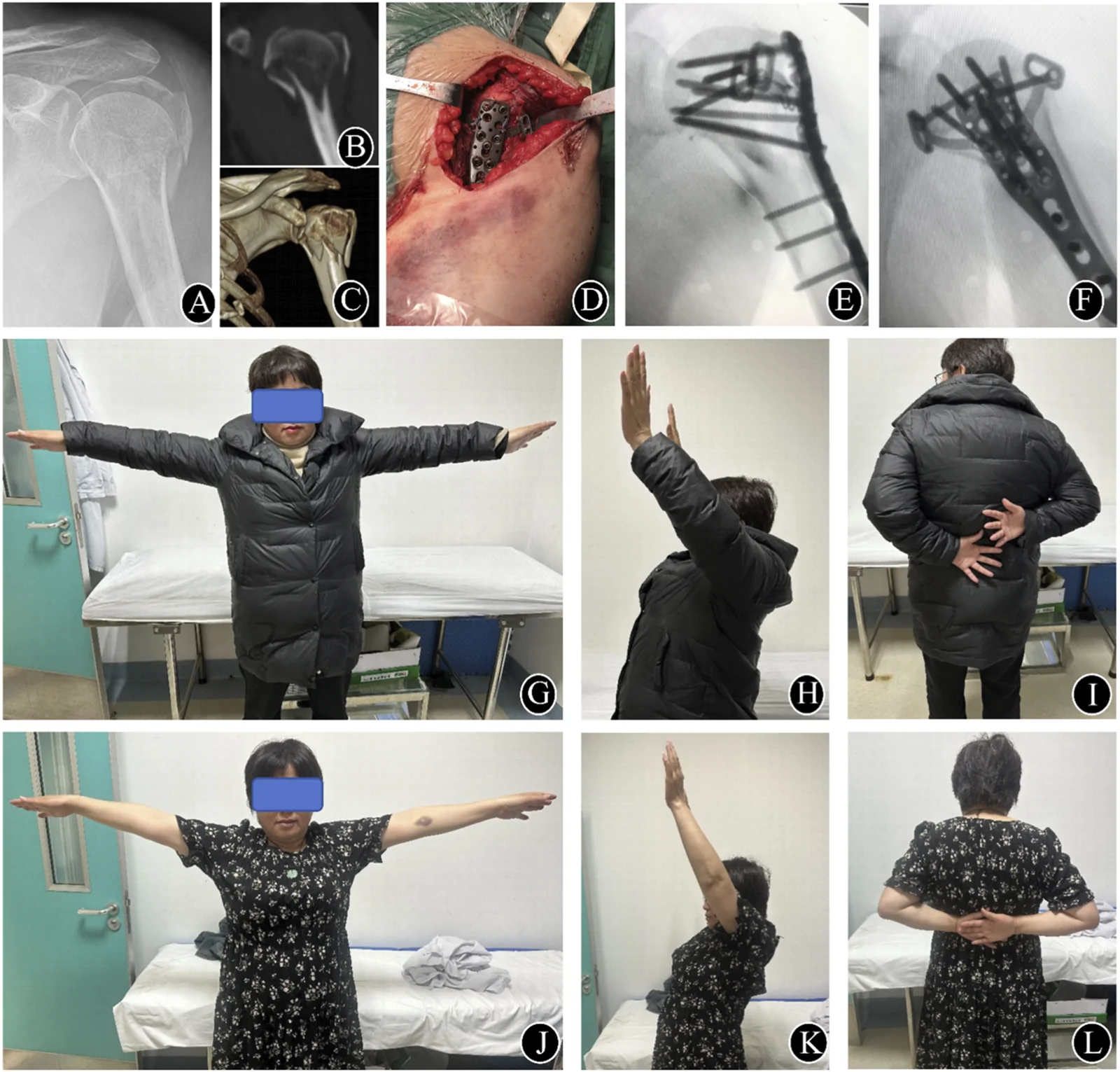
Case 1: dual-wing plate (48-year-old woman, Neer four-part fracture). **(A–C)** Preoperative radiographs and CT; **(D–F)** intraoperative fluoroscopy; **(G–I)** 3-month follow-up; **(J–L)** 6-month follow-up with maintained reduction and full functional recovery.

Case 1 (Group A — dual-wing plate): A 48-year-old woman sustained a Neer four-part proximal humeral fracture in a low-energy fall (Figures 7A–C). Bilateral wing fixation was performed, with an individualized calcar screw trajectory directed toward the posteromedial trabecular column; intraoperative fluoroscopy confirmed anatomical reduction (Figures 7D–F). Recovery was progressive, with substantial return of active shoulder motion by 3 months (Figures 7G–I). At 6 months the reduction was maintained and active shoulder motion had returned to normal in all planes (Figures 7J–L).

**FIGURE 8:**
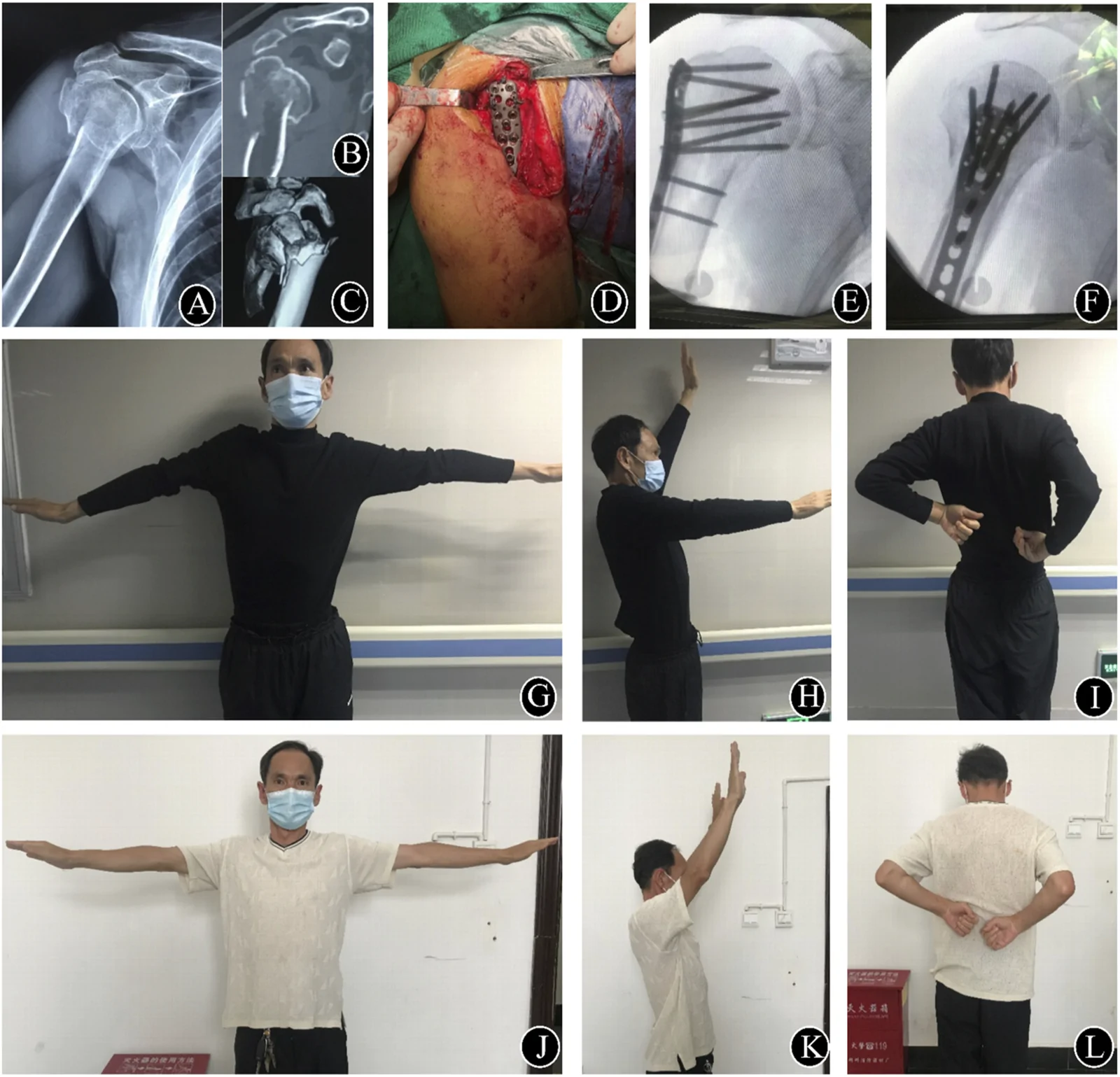
Case 2: PHILOS plate (50-year-old man, Neer four-part fracture). **(A–C)** preoperative radiographs and CT; **(D–F)** intraoperative fluoroscopy; **(G–I)** 3-month — restricted motion; **(J–L)** 6-month — substantial functional recovery.

Case 2 (Group B — PHILOS plate): A 50-year-old man sustained a right-sided Neer four-part fracture in a road traffic accident (Figures 8A–C). Standard PHILOS fixation was performed with supplementary suture tuberosity repair (Figures 8D–F). At 3 months, active motion remained clearly restricted (Figures 8G–I). By 6 months, ROM had recovered substantially (Figures 8J–L).

## 4 Discussion

The central finding of this study is that the dual-wing plate does not reduce stress or displacement at the bone level compared with PHILOS — the real data show these values are essentially identical across all four abduction angles. What the dual-wing plate achieves, and what the FEA demonstrates clearly, is a qualitative redistribution of where stress concentrates within the implant. In the PHILOS model, the highest von Mises stress occurred consistently at the proximal calcar screw-plate junction across every loading angle. In the dual-wing model, the peak shifted to the wing-main plate interface. This is not a trivial distinction: the calcar screw junction sits immediately adjacent to the humeral articular surface, and failure there — the classic cut-out and varus collapse pattern documented repeatedly in published series — directly compromises the joint (Varga et al., 2020; Liu et al., 2024). The wing-plate junction, by contrast, lies over the tuberosity cortex, away from the articular surface and accessible for revision if needed.

The pattern of stress divergence across the four angles deserves careful reading. At 0° and 30°, peak plate stress was nearly equal between constructs (PHILOS 42.9 and 59.4 MPa; dual-wing 43.3 and 60.6 MPa). As abduction increased to 60° and 90°, the dual-wing plate carried progressively higher peak stresses than PHILOS (82.3 vs. 67.2 MPa at 60°; 108.7 vs. 68.4 MPa at 90°). This is expected behavior: as supraspinatus, infraspinatus, and teres minor increase their force output with arm elevation, those forces transmit through the wing extensions directly to the wing-plate junction. The wings are acting as designed — they are the load-bearing interface between the tuberosity and the main plate body. Whether this junction can tolerate these stress levels over a fatigue loading history is a question that requires dedicated cyclic mechanical testing beyond the scope of this FEA, but one that informed the implant design to use a snap-lock reinforced connection rather than a simple screw-hole interface.

The clinical advantage of the dual-wing plate — earlier motion, better early function, and zero tuberosity complications — flows from a different mechanism than pure stress reduction. When the wing extensions provide direct rigid multi-screw fixation of each tuberosity fragment, the surgeon’s intraoperative assessment of construct stability changes. In the PHILOS group, the reliance on supplementary sutures creates inherent mechanical uncertainty: suture pull-through, knot slippage, and the flexibility of soft tissue repair mean the surgeon cannot be as confident about resistance to early tuberosity displacement. Voigt et al. (Voigt et al., 2009) showed biomechanically that additive sutures add little to PHILOS fracture stabilization, and Badman et al. (Badman et al., 2011) reported that rotator cuff complications persisted even with suture augmentation. This uncertainty influences rehabilitation decisions. In Group A, the surgeon initiated active motion 3.7 days earlier on average — driven by intraoperative clinical assessment rather than a protocol change — and this difference had downstream effects on pain at 1 month, CMS at one and 2 months, and abduction and external rotation ROM at early timepoints. The groups converged by 6 months, consistent with the interpretation that early fixation confidence, not long-term structural superiority, was the operative variable.

The mechanobiological implications connect this work to the journal’s focus on immunometabolic aspects of musculoskeletal repair. Tuberosity malunion does not simply represent a mechanical failure; it sustains an extended inflammatory state at the fracture interface. Delayed union prolongs M1-dominant macrophage activation, with elevated TNF-α, IL-6, and RANKL, suppressing osteoblast differentiation and driving pathological bone resorption (Claes et al., 2012; Raggatt and Partridge, 2010; Schlundt et al., 2018). The trend toward fewer delayed unions in Group A (10.5% vs. 29.2% at >12 weeks) suggests that more stable tuberosity fixation may shorten this inflammatory window, though the sample size precludes definitive conclusions. No tuberosity nonunions or malunions occurred in Group A; three occurred in Group B. Given the established dependence of Constant-Murley score on tuberosity healing quality (Kettler et al., 2006), the absence of these complications in Group A is the most directly clinically meaningful outcome of this series.

The calcar screw stress comparison carries direct implications for PHILOS failure mechanisms. Published series have consistently identified calcar screw cut-out as the leading hardware complication in proximal humeral locking plate fixation (Varga et al., 2020; Liu et al., 2024). Gardner et al. (Gardner et al., 2008) established that inadequate inferomedial cortical support is among the strongest predictors of varus collapse. The omnidirectional calcar screw of the dual-wing plate addresses this by allowing the surgeon to aim toward the densest posteromedial trabecular bone regardless of native neck-shaft angle. No calcar screw cut-outs occurred in either group in this series, but the stress redistribution seen in FEA — where calcar region stress in the dual-wing plate remained at levels seen at 0° and 30° even as the wing-plate junction stress escalated — provides a structural rationale for reduced cut-out risk over a larger patient cohort and longer follow-up.

A concern sometimes raised about rigid tuberosity fixation is whether it might prevent the interfragmentary micromotion needed for callus formation, or predispose to adhesive capsulitis by constraining early motion. The clinical data argue against both. Group A patients — those with the most rigid fixation — moved earliest, reported least early pain, and none developed persistent stiffness. This is consistent with the established principle that it is prolonged immobilization, not stable fixation, that drives periarticular fibrosis (Perren, 2002).

The dual-wing plate was not more surgically demanding: operative time and blood loss were equivalent between groups. The modular wing design allows deployment of one or both wings according to fracture configuration. The experienced surgeons in the PHILOS group ensure that observed functional differences reflect implant mechanics rather than a learning curve effect.

Limitations should be stated plainly. The retrospective single-center design with 43 patients limits statistical power; several complication differences, though clinically meaningful in direction, did not reach significance. Implant allocation was not randomized. No bone mineral density data were collected. The FEA model uses isotropic linear elastic materials and static loads — simplifications that capture the principal stress patterns but do not reproduce viscoelastic, cyclic, or dynamically varying *in vivo* conditions. The wing-plate junction, identified as the highest-stress site in the dual-wing model at higher abduction angles, has not been subjected to fatigue analysis in this work. Follow-up of 12 months is insufficient for full AVN assessment. Recent long-term data comparing two locking-plate constructs for three- and four-part fractures report clinical and complication outcomes at a minimum of 6 years (Conteduca et al., 2026); our 12-month follow-up cannot address such long-term differences. A prospective randomized trial, powered on tuberosity complication rates as the primary endpoint, is the appropriate next step.

## 5 Conclusion

The principal biomechanical finding of this FEA study is a qualitative redistribution of peak implant stress—from the calcar screw-plate junction in the PHILOS plate to the wing-main plate interface in the dual-wing plate—without reducing overall bone-level stress or displacement. In our small retrospective cohort, this stress redistribution, together with direct rigid multi-screw fixation of tuberosity fragments, was associated with earlier initiation of passive pendulum exercises and lower early pain scores. However, the absence of screw cut-out in either group prevents any conclusion regarding reduced cut-out risk, and the observed trends in tuberosity complications, while clinically encouraging, did not reach statistical significance. The elevated stresses at the wing-plate junction at higher abduction angles warrant dedicated cyclic fatigue testing. These findings are hypothesis-generating and require confirmation in adequately powered, prospective randomized trials with longer follow-up before any claim of clinical superiority can be established.

## Data Availability

The original contributions presented in the study are included in the article/Supplementary Material, further inquiries can be directed to the corresponding authors.
