# Supplementary material for: Biomechanical and clinical comparison of a novel dual-wing locking plate versus the PHILOS plate for neer three- and four-part proximal humeral fractures: a finite element analysis combined with a retrospective cohort study
Source: Front Bioeng Biotechnol. 2026 Aug 19;14:1865863. doi: 10.3389/fbioe.2026.1865863 (PMC13533890; doi:10.3389/fbioe.2026.1865863)
Supplement: Supplementary file 1 [file Supplementaryfile1.docx]

**Supplementary Material**

*Biomechanical and Clinical Comparison of a Novel Dual-Wing Locking Plate Versus PHILOS Plate for Neer Three- and Four-Part Proximal Humeral Fractures*

## Supplementary Table S1. FEA material properties and mesh parameters (Feerick et al., 2013; Jabran et al., 2019).

| **Component** | **Elastic modulus (MPa)** | **Poisson's ratio** | **Elements (n)** | **Nodes (n)** |
| --- | --- | --- | --- | --- |
| Cortical bone | 20,000 | 0.30 | 121,366 | 32,116 |
| Cancellous bone | 100 | 0.26 | 69,671 | 15,653 |
| Scapula | 9,000 | 0.30 | 98,215 | 24,536 |
| Ligaments | 150 | 0.30 | 315 | 534 |
| Ti6Al4V (plates & screws) | 120,000 | 0.30 | 17,633 | 5,705 |

All materials assigned as isotropic linear elastic. Scapula properties as humeral cortical bone. Contact conditions: screw–bone and plate–screw interfaces bonded; dual-wing extension–main plate frictionless; fracture interfaces frictionless; cortical–cancellous interface bonded. Ligaments modeled as tension-only circular beam elements (diameter 2 mm).

## Supplementary Table S2. Rotator cuff loading parameters at each abduction angle (Keating et al., 1993; Apreleva et al., 2000).

| **Abduction angle** | **Supraspinatus (N)** | **Subscapularis (N)** | **Infraspinatus (N)** | **Teres minor (N)** |
| --- | --- | --- | --- | --- |
| 0° | 100 | 60 | 30 | 20 |
| 30° | 120 | 80 | 60 | 40 |
| 60° | 150 | 100 | 80 | 50 |
| 90° | 180 | 120 | 100 | 60 |

Infraspinatus and teres minor listed individually; their resultant was applied as a single combined vector at 129° clockwise from the Y-axis. Supraspinatus applied at 172°; subscapularis at 122°, following the loading directions of Apreleva et al. (2000). Loading directions based on the glenoid-centered local coordinate frame (X-axis: scapular coronal plane; Y-axis: horizontal plane; Z-axis: sagittal plane).

**Supplementary Figure S1. Design illustration of Dual-Wing Locking Plate**

**Supplementary Figure S1.** *Schematic of the dual-wing locking plate.* Omnidirectional calcar screw: A and B show the ball-head omnidirectional locking mechanism; C–E show the adjustable range of the screw axis in the coronal and sagittal planes (maximum ±15°), allowing individualized medial-column support. Dual-wing design: F show the snap-lock interfaces on both sides of the main plate; G shows the front and back views of the assembled wing mini-plate and main plate.

## Supplementary Figures S2–S5. Locking screw finite element results at all abduction angles.

The following four figures present locking screw von Mises stress and displacement results from the finite element analysis at each of the four abduction angles tested (0°, 30°, 60°, 90°). These data complement Figures 2–5 in the main manuscript, which present plate body and bone fragment results. For each angle, stress and displacement contour maps are shown as a 2×2 panel: panels A and B show von Mises stress (MPa) for the PHILOS and dual-wing constructs respectively; panels C and D show resultant displacement (mm) for the PHILOS and dual-wing constructs respectively. Across all angles, the PHILOS model shows stress concentration at the calcar screw tip, which becomes more pronounced at 60° and 90° abduction — consistent with the clinical risk of calcar screw cut-out during early active rehabilitation.

**Supplementary Figure S2.** *Locking screw results at 0° abduction (neutral position).* A: PHILOS screw von Mises stress (MPa); B: dual-wing screw von Mises stress (MPa); C: PHILOS screw displacement (mm); D: dual-wing screw displacement (mm). Rotator cuff forces at this position are at their lowest values (supraspinatus 100 N, subscapularis 60 N, infraspinatus 30 N, teres minor 20 N); screw-level stresses are correspondingly modest in both constructs.

**Supplementary Figure S3.** *Locking screw results at 30° abduction.* A: PHILOS screw stress (MPa); B: dual-wing screw stress (MPa); C: PHILOS screw displacement (mm); D: dual-wing screw displacement (mm). Increasing supraspinatus activation (120 N) begins to load the greater tuberosity-side screws preferentially. A local stress concentration at the PHILOS calcar screw tip begins to emerge at this angle.

**Supplementary Figure S4.** *Locking screw results at 60° abduction.* A: PHILOS screw stress (MPa); B: dual-wing screw stress (MPa); C: PHILOS screw displacement (mm); D: dual-wing screw displacement (mm). At 60°, peak rotator cuff forces produce the highest absolute screw stresses across both constructs. The PHILOS calcar screw tip stress concentration is prominent at this angle, consistent with the clinical observation that screw cut-out events tend to occur during active elevation rather than at rest.

**Supplementary Figure S5.** *Locking screw results at 90° abduction.* A: PHILOS screw stress (MPa); B: dual-wing screw stress (MPa); C: PHILOS screw displacement (mm); D: dual-wing screw displacement (mm). Full rotator cuff co-activation at 90° places maximum torsional and shear demand on the implant-bone interface. The PHILOS calcar screw tip stress concentration reaches its highest level at this angle. The dual-wing screw assembly shows lower focal stress, attributable to load-sharing across the additional wing extension screws.

## Supplementary Note. Statistical approach for FEA comparisons.

Finite element analysis in this study was performed as a deterministic computational simulation. Between-group (dual-wing vs. PHILOS) comparisons of peak stress and displacement values reflect single-run outputs from fully specified geometric and material models; they do not carry statistical uncertainty in the conventional sense. The values reported in the main manuscript text represent direct readouts from the ANSYS 2022R1 color-bar legend maximum values for each simulation condition. No post-hoc statistical testing was applied to the FEA outcome variables, consistent with standard practice in deterministic computational biomechanics studies.
